# Real-time imaging of transcriptional feedback in nonsense-mediated mRNA decay

**DOI:** 10.1101/2025.05.20.655238

**Published:** 2025-05-21

**Authors:** Hanae Sato, Md. Dobirul Islam, Tamoghna Das, Robert H. Singer

**Affiliations:** 1 WPI Nano Life Science Institute (NanoLSI), Kanazawa University, Kanazawa, Ishikawa, Japan; 2 Department of Cell Biology, Albert Einstein College of Medicine, 1300 Morris Park Ave, Bronx, NY 10461, USA

## Abstract

Nonsense-mediated mRNA decay (NMD) is a translation-coupled mRNA decay pathway triggered by a premature termination codon (PTC). While in-frame stop codons are typically defined by cytoplasmic ribosomes, unexpected changes in transcription have been reported in genes containing PTCs. This observation suggests the possibility of PTC detection at the transcription site, which has not been thoroughly investigated with high temporal and spatial resolution. Here we utilize a real-time imaging approach to simultaneously detect transcription sites expressing wild-type or NMD-targeted β-globin reporter genes in the same cell. Our data indicates a dynamic change in the transcription of PTC-containing β-globin mRNA that depends on translation, NMD, and nuclear protein import, supporting the existence of rapid transcriptional feedback following NMD in the cytoplasm. This study establishes a robust temporal link between cytoplasmic mRNA decay and nuclear transcription.

## One-Sentence Summary

High temporal and spatial detection of transcriptional feedback in Nonsense-mediated mRNA decay.

The flow of gene expression from transcription to translation, commonly known as the central dogma, is typically portrayed as a unidirectional process. However, several studies have provided evidence of crosstalk between cytoplasmic mRNA decay and transcription (1). Such crosstalk can occur through mechanisms that alter the rates of mRNA decay and transcription, thereby regulating cellular protein concentrations (2–4). Notably, this phenomenon has been observed in mammalian cells during viral infection induced by the gamma-herpesvirus endonuclease SOX (5), and in the context of nonsense-mediated mRNA decay (NMD)(6–9).

In contrast to the global regulation of transcription rates, the transcriptional changes of PTC-containing transcripts are sequence-specific, with these transcripts modulating the expression of their own or homologous genes. Specifically, PTC-containing pre-mRNAs—but not those with missense or frameshift mutations—have been found to accumulate at the transcription sites of immunoglobulin (Ig) μ and T cell receptor (TCR) β genes (6, 10). This accumulation results from transcriptional retention rather than increased transcriptional activity.

Further studies have demonstrated that the deleterious effects of nonsense mutations can be mitigated by the upregulation of homologous genes, induced by cytoplasmic NMD (8, 9). These findings emphasize the role of transcriptional regulation via nonsense-coding transcript degradation in genetic compensation, underscoring the biological significance of nonsense-associated transcriptional regulation.

Moreover, in cells carrying heterozygous disease-associated nonsense mutations, the levels of normal mRNA expressed from the wild-type allele were found to be reduced (11, 12), suggesting that nonsense-associated transcriptional alterations may influence the severity of phenotypic expression in heterozygous nonsense mutations. Despite these insights, the molecular mechanism underlying transcriptional regulation by cytoplasmic mRNA decay remains unknown.

**Fig. 1.**
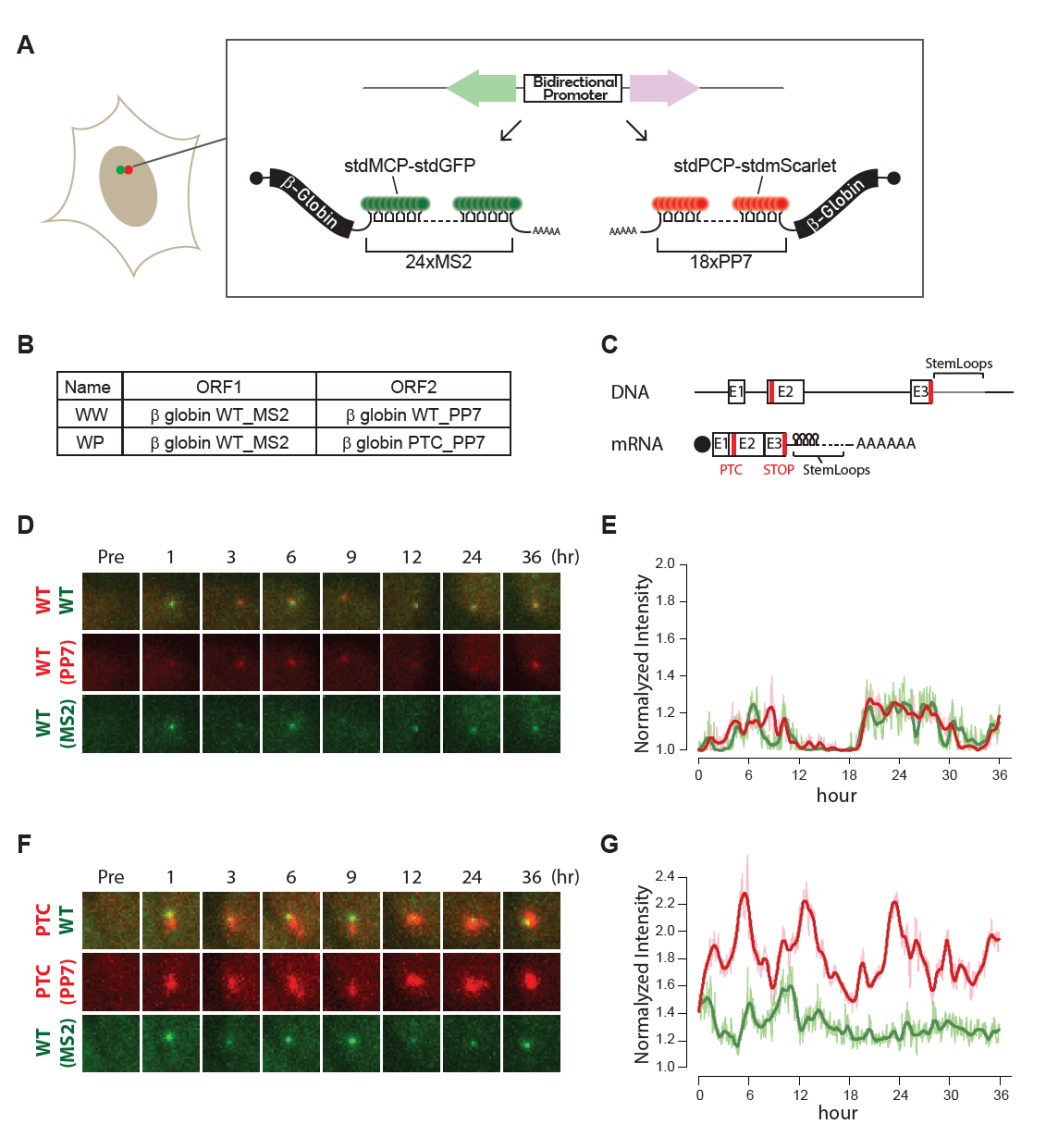
Simultaneous real-time tracking of transcription activities at the NMD reporter with and without PTC. (A) Schematic of PonA-inducible bi-directional promoter expressing NMD reporter β-globin genes. The transcripts expressed from either direction of the promoter contain MS2 or PP7 sequences in the 3’ UTR, which are labeled with stdMCP-stdGFP or stdPCP-stdmScarlet. 24 or 18 repeats of MS2 or PP7, resulting in a similar 3’UTR length, were used. (B) The table shows the mRNAs expressed from the bi-directional promoter of each construct (WW; Wild-type and Wild-type or WP; Wild-type and PTC-containing β-globin). WT; wild-type, PTC; premature termination codon. (C) Structures of the NMD reporter Gl (DNA; upper and mRNA; lower) containing a PTC at position 39 (PTC). Horizontal lines represent introns, 5’-UTR and 3’-UTR, while boxes represent each of the three Gl exons (E1–3) joined by splicing-generated exon-exon junctions. Black dot represents the cap structure; red lines indicate termination codons; STOP represents normal termination codon; AAAAAA indicates poly(A) tail. (D-G). Images of transcription sites before (Pre) and after (3–36 hours) transcription induction by PonA. The image size of each transcription site shown here is 7 × 7 μm^2^. (E&G) Normalized intensity of transcription sites was plotted. Each transcription site was imaged every 2 minutes, and the intensities of transcription sites were detected using TrackMate (1). The mean intensity of the transcription site was normalized by the mean nuclear intensity at each time point. The mean intensity of the transcription site was normalized by the mean nuclear intensity at each time point. Normalized intensity at each transcription site of Gl-MS2 (green) or Gl-PP7 (pink) was smoothed and fitted using the lowess method (Gl-MS2 (dark green) or Gl-PP7 (red)) in R.

To investigate the relationship between transcriptional activity and cytoplasmic NMD, we developed a real-time imaging system to monitor the transcriptional dynamics of an NMD reporter gene in living cells. This system utilizes a ponasterone A (ponA)-inducible bidirectional reporter that co-expresses both PTC-free and PTC-containing β-globin mRNAs, each tagged with MS2 or PP7 in their 3’ UTRs (13) (Fig. 1A). By enabling simultaneous detection of transcription in living cells, our system allows for precise examination of the dynamic transcriptional response during mRNA decay with high spatiotemporal resolution.

To establish a control, we expressed the wild-type β-globin gene in both directions within a single cell (Fig. 1B; WW: Wild-type and Wild-type). For the NMD reporter, we expressed wild-type and PTC-containing β-globin genes in opposite orientations within the same cell (Fig. 1B&C, WP: Wild-type and PTC). U2OS cells stably expressing WW or WP were generated using the Flp-In system, enabling us to compare transcriptional activity at the same chromatin locus while minimizing variability associated with transcription at different chromatin locations (13, 14). Given that the insertion of multiple stem loops in the 3′ UTR could potentially induce NMD (15), we assessed the impact of a 3′ UTR extension on mRNA stability by inserting stem loops and knocking down UPF1 using shRNA. The levels of endogenous UPF1 and control β-actin mRNA were measured by RT-qPCR, confirming successful knockdown of UPF1 (Figure S1). Additionally, comparison of reporter mRNA levels containing MS2 or PP7 stem loops in the WW construct between cells expressing control shRNA and shRNA against UPF1 indicated that the insertion of these stem loops did not trigger NMD in this system (Fig. S1).

To simultaneously examine transcriptional dynamics at wild-type and PTC-containing mRNA transcription sites, we imaged transcriptional activity in cells expressing WW (Fig. 1D&E) or WP (Fig. 1F&G) every two minutes in real-time. Following transcription induction with PonA, we quantified fluorescence intensities at transcription sites using TrackMate (16). The analytical pipeline used to identify transcriptional bursts (peaks) in Figure 1 is outlined in Figure S2A. We then analyzed the average intensities at peaks in multiple cells expressing WW (Fig. S2B) or WP (Fig. S2C). Notably, we observed enhanced transcriptional bursts in WP-expressing cells (Fig. 1F&G, Fig. S2C, movie S2) compared to WW-expressing cells (Fig. 1D&E, Fig. S1B, movie S1) over a 36-hour period.

**Fig. 2.**
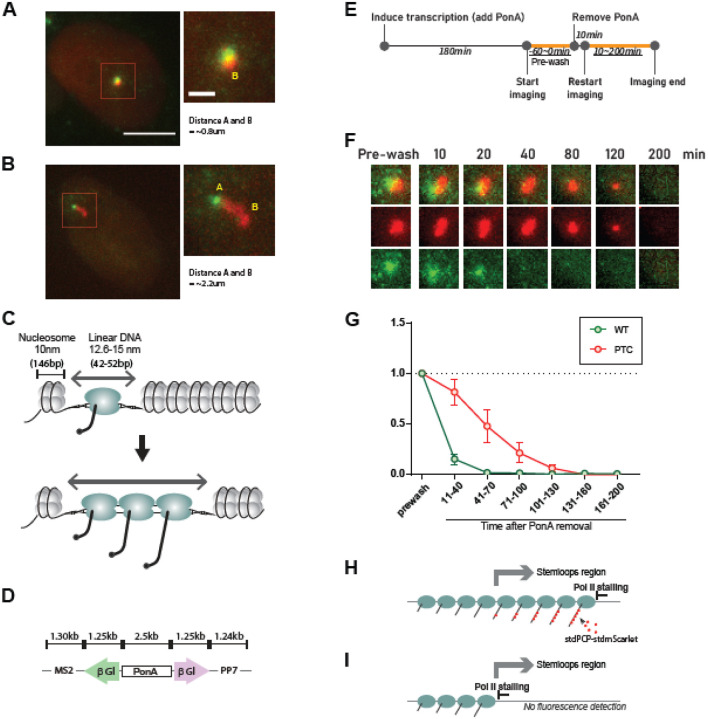
Simultaneous detection of transcription sites with and without a PTC reveals that enlargement of the transcription site is due to retention of PTC-containing nascent transcripts. (A) Image of a transcription site expressing wild-type β-globin mRNA in both orientations. (B) Images of transcription sites expressing either wild-type or PTC-containing β-globin mRNA from each orientation of the bidirectional promoter. Scale bar = 10 μm (left). Insets show higher magnification (scale bar = 2 μm). The region outlined in red is shown on the right of each image. (C) Schematic representation of stretched chromatin structure due to occupancy by elongating RNA polymerase II. (D) Approximate length of the reporter construct. (E) Detection of transcription sites expressing the WP construct before and after removal of the transcription inducer. Timeline indicates transcriptional activation via PonA addition followed by its removal. Orange lines and underlined time points indicate the duration of real-time imaging. (F) Images of transcription sites before (Pre-wash) and after PonA removal (10–100 minutes). Each transcription site image represents a 7 × 7 μm^2^ field. (G) Quantification of transcriptional activity from the WP construct before (Pre-wash) and after PonA removal (10–100 min). Dots represent the mean normalized intensity of transcription sites across 11 cells. Error bars represent the standard deviation within the cell population. Statistical analysis was performed using an unpaired, two-tailed *t*-test in GraphPad Prism. P values for the 11–40 min, 41–70 min, 71–100 min, 101–130 min, 131–160 and 161–200 min time windows were 0.000407, 0.005464, and 0.000291, respectively. (H, I) Schematics illustrating transcriptional stalling of elongating RNA polymerase II at the end (H) or in the middle (I) of the gene.

The transcription site expressing wild-type mRNA appeared as a fluorescent spot (Fig. 2A). In contrast, the transcription site for PTC-containing mRNA exhibited dynamic changes and was frequently observed as a stretched or enlarged structure (Fig. 2B). The length of the stretched transcription site roughly corresponded to the estimated linear length of the gene in the absence of chromatin condensation (17) (Fig. 2BCD). This PTC-specific transcriptional enlargement was also observed in another NMD transcript, the PTC-containing Ig μ mini-gene (Fig. S3), providing additional evidence for the role of PTCs in driving transcriptional enlargement.

The elongated transcription site could result from increased transcription initiation, frequent pausing, slower RNA polymerase progression, or impaired transcription termination, all of which may lead to stalled RNA polymerases (Fig. 2C). To investigate this further, we performed a pulse-chase assay, where transcription was induced with PonA which was then subsequently removed (Fig. 2E). Our results showed that while the fluorescence intensity at the transcription site of wild-type mRNA disappeared after PonA removal, the fluorescence intensity at the transcription site of PTC-containing mRNA persisted for over 100 minutes after inducer removal (Fig. 2F&G). This suggests that transcription had stalled at the PTC-containing β-globin gene, consistent with previous reports (6, 10). This likely represents stalled RNA polymerase II at the end of the gene, downstream of the stem loops (Fig. 2H), as transcripts from ongoing transcription upstream of the stem loops would not be visible (Fig. 2I).

PTC-dependent transcriptional enlargement raises the question of how a premature termination codon (PTC) could influence the transcription site before mRNA is exported to the cytoplasm, given that current biological evidence suggests PTCs are recognized only by translating ribosomes in the cytoplasm. Possible mechanisms include recognition of the PTC during transcription or the presence of an unknown feedback signal from the cytoplasm to the transcription site following PTC recognition. To explore this, we examined transcription sites immediately after PonA induction, at which point mRNAs have not yet been exported or translated. Fluorescence intensities at transcription sites were measured every 2 minutes for the first 2.5 hours after induction in cells expressing either the WW (Fig. S4A) or WP (Fig. S4B) construct, and the average intensity of the transcription sites was calculated. The data showed that transcriptional enlargement occurred in WP-expressing cells, but not in WW-expressing cells, beginning approximately 1 to 1.5 hours after transcription initiation. This suggests that the enlargement is not triggered immediately by PTC recognition during transcription. Instead, the observed delay of 1 to 1.5 hours may reflect the time required for downstream processes such as mRNA export, translation, nonsense-mediated decay (NMD), and subsequent feedback to the transcription site.

**Fig. 3.**
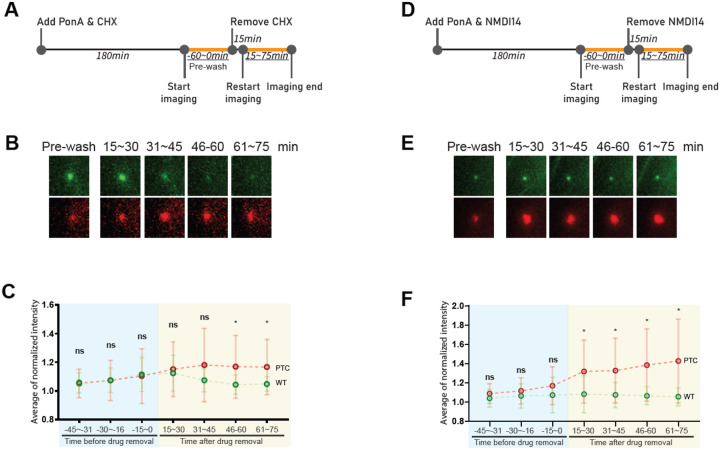
Rapid feedback of transcription preceded by translation and NMD. (A) Timeline of transcription induction by supplementation of PonA with CHX (100 μg/ml) followed by drug removal. Orange lines and underlined times indicate the time duration for real-time imaging. (B) Images of transcription site before (Pre-wash) and after removal of CHX (15–75 minutes). The image size of each transcription site shown here is 8 × 8 μm^2^. (C) Detection of transcription sites expressing the WP construct before (Pre-wash) and after removal of CHX. Single dots denote the mean normalized intensity of wild-type (Green) or PTC-containing β-globin transcription sites (Red) during the indicated time duration from 13 cells. Statistical analysis was performed using an unpaired, multiple t-test with the Mann–Whitney test (without correction) in GraphPad Prism. P values for the 46–60 min and 61–75 min time windows were 0.008204 and 0.016083, respectively. (D) Timeline of transcription induction by supplementation of PonA with the NMD inhibitor, NMDI14, followed by the removal of NMDI14 (40 μM). Orange lines and underlined times indicate the time duration for real-time imaging. (E) Images of transcription site before (Pre-wash) and after removal of NMDI14 (15–75 minutes). The image size of each transcription site shown here is 8 × 8 μm^2^. (F) Detection of transcription sites expressing the WP construct before and after removal of NMDI14. Single dots denote the mean normalized intensity of wild-type (Green) or PTC-containing β-globin transcription sites (Red) during the indicated time duration from 14 cells. Error bars represent the standard deviation within the cell population. Statistical analysis was performed using an unpaired, multiple t-test with the Mann–Whitney test (without correction) in GraphPad Prism. P values for the 1–15, 16–30, 31–45, 46–60 min time windows were 0.023773 and 0.007530, 0.003374, 0.000672, respectively.

We next investigated whether the transcriptional effect is translation-dependent, as translation is required for PTC recognition by ribosomes and subsequent NMD activation in the cytoplasm. To test this, we used the translation inhibitor cycloheximide (CHX). First, we confirmed that CHX had no effect on transcription sites in cells expressing the WW construct (Fig. S5A–B). We then examined whether translation inhibition affected the transcriptional enlargement in PTC containing transcription sites. Our results showed that transcriptional enlargement was observed without CHX (Fig. S5C) but not in CHX-treated PTC containing transcription site (Fig. S5D). Furthermore, we found that transcriptional enlargement was abolished during CHX treatment but reappeared upon CHX removal (Fig. 3A–C), indicating that the feedback to the transcription site is translation-dependent.

To further explore the role of NMD, we used the NMD inhibitor NMDI14, which interfere SMG7-UPF1 interactions (18). Similar to CHX, NMDI14 treatment blocked transcriptional enlargement, and its removal led to a rapid reappearance of the enlarged transcription site (Fig. 3D–F), further supporting that this phenomenon is NMD-dependent. Importantly, this effect was not due to medium replacement itself, as control experiments using DMSO-containing medium instead of NMDI14 had no impact on the transcription site (Figure S6).

The alteration of the transcription site was specific to the presence of the PTC, which resulted from a single nucleotide substitution in the wild-type gene. This sequence-specific effect suggests the existence of a mechanism that discriminates between mutated alleles in DNA or nascent mRNA at the transcription site. One possible explanation for the PTC-specific effect is the recognition of the allele by mRNA degradation fragments, which result from endonucleolytic cleavage of the mRNA near the PTC by SMG6 (19, 20). These RNA fragments could potentially anneal with the DNA where the PTC-containing transcripts are originally produced. To test the role of mRNA fragments in transcriptional feedback, we introduced self-endonucleolytic mRNA cleavage by inserting a hammerhead ribozyme structure between the β-globin coding region and PP7 stem loop sequence in the WW construct (Fig. S7A; WH: Wild-type and Hammerhead). We generated a single-locus integrated WH construct in the PonA cell line and monitored transcriptional activity every two minutes for 36 hours. Unexpectedly, we observed comparable transcription activities (without enlargement) in WH-expressing cells, indicating that mRNA degradation fragments were not sufficient to trigger transcriptional feedback (Fig. S7A–B). However, we cannot exclude the possibility that the self-endonucleolytic mRNA cleavage by the hammerhead ribozyme failed to trigger transcriptional feedback due to insufficient cleavage efficiency—approximately 50% compared to the wild-type construct without the hammerhead ribozyme insertion, as confirmed by RT-qPCR (Fig. S7C).

Since self-endonucleolytic mRNA cleavage with the hammerhead ribozyme did not activate transcriptional feedback, it is likely that feedback is NMD-dependent, or RNA-binding proteins involved in mRNA decay are required for this mechanism. To investigate this possibility, we tested whether inhibition of nuclear protein import using the transport receptor importin-β inhibitor, importazole (21), reduces the transcriptional enlargement of the PTC-containing β-globin. We induced transcription under CHX treatment, then replaced CHX with fresh medium containing 20 μM PonA and 50 μM importazole (Fig. S8A–B). We monitored the transcription sites before and after the removal of CHX and the addition of importazole. The imaging data showed that the nuclear import inhibitor reduced the transcriptional enlargement of the PTC-containing transcript (Fig. S8A–B), suggesting the involvement of a protein or RNP regulator.

**Fig. 4.**
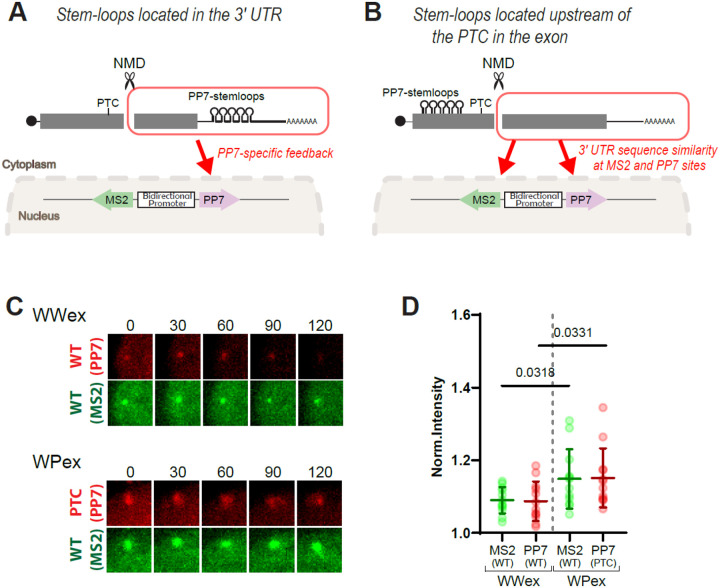
Insertion of stem loops downstream of the PTC within an exon enables transcriptional feedback in both alleles. (A) Schematic representation of stem-loop insertion into the 3’UTR. The 3’ fragments generated by endonucleolytic cleavage during NMD provide feedback only to the PP7 allele due to sequence similarity in the 3’UTR. (B) Schematic representation of stem-loop insertion downstream of the PTC within an exon and the expected feedback mechanism. The 3’fragments generated by NMD cleavage can provide feedback to both the MS2 and PP7 alleles due to the shared sequence in the 3’UTR. (C) Representative images of transcription sites using a reporter with stem loops inserted downstream of the PTC within an exon. After overnight induction of transcription with PonA, time-lapse imaging was conducted for two hours. Each image shows an 8 × 8 μm^2^ region centered on the transcription site. (D) Detection of transcription sites in cells expressing either the WWex or WPex construct, both containing stem loops inserted into the exon. Each dot represents the mean normalized intensity of transcription sites for wild-type (green) or PTC-containing (red) β-globin mRNA, measured over the indicated time period in 11 cells. Error bars indicate the standard deviation. Statistical analysis was performed using an unpaired, two-tailed t-test in GraphPad Prism.

Notably, transcriptional feedback was observed exclusively at sites expressing PTC-containing transcripts, but not at sites expressing the wild-type transcripts, which were transcribed from the opposite side of a bi-directional promoter in this reporter system. In this setup, the key differences between the wild-type and PTC-containing transcripts lie in the presence of the PTC and the positioning of MS2 or PP7 stem loops within the 3’UTR. We speculate that the limited sequence similarity in the 3’UTR—particularly at the sites of stem loop insertion—may underlie the observed allele-specific feedback. Specifically, the PTC-containing transcripts generate a PP7-tagged 3’fragment that is absent from the wild-type, potentially driving feedback at the transcription site (Fig. 4A). Based on this, we hypothesized that transcriptional feedback could occur at both wild-type and PTC-containing sites if the 3’fragments generated during NMD share a common sequence context. To test this, we inserted stem loops into an exon downstream of the PTC (WWex and WPex constructs, Fig. 4B), where cleavage near the PTC is expected to generate 3’fragments with similar downstream sequences (19, 20). Remarkably, imaging analysis revealed transcriptional feedback at both wild-type (no PTC) and PTC-containing transcription sites under these conditions (Fig. 4C–D), supporting our hypothesis.

**Fig. 5.**
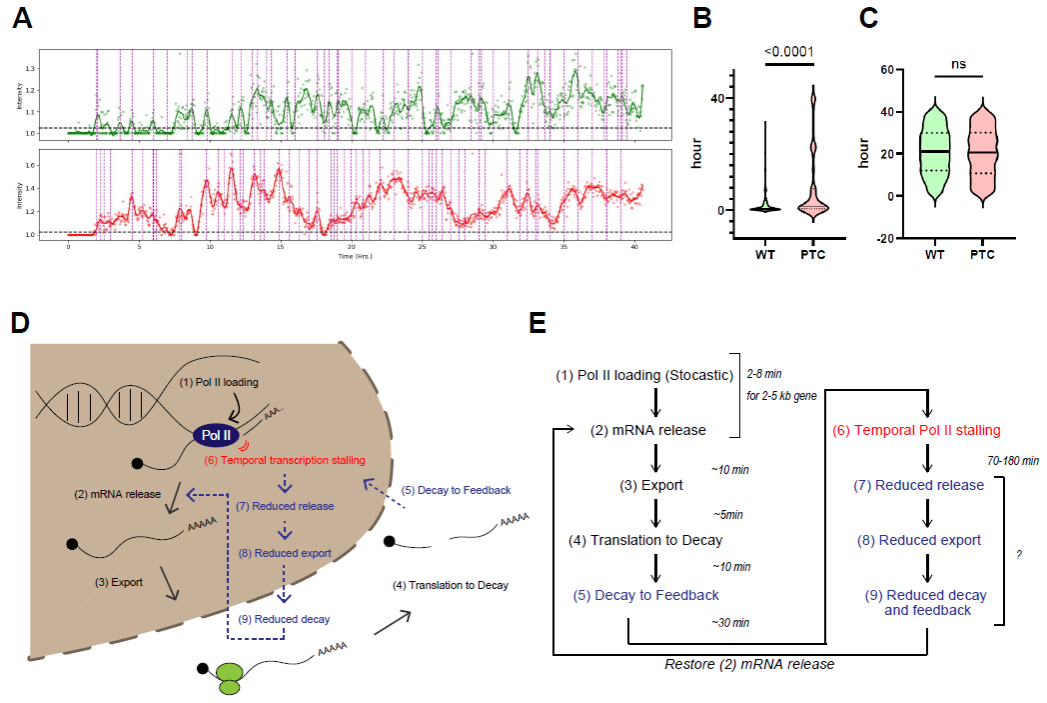
Model of feedback loop in NMD. (A) Example of transcription site (TS) intensity transition. Green and red lines represent the TS intensity of the wild-type and PTC-containing gene, respectively. (B) Violin plot showing the frequency of transcriptional bursting per hour. “ns” indicates not significant. Dotted lines represent quantiles; thick lines indicate medians. Statistical analysis was performed using an unpaired, two-tailed t-test in GraphPad Prism. (C) Violin plot showing the duration of the transcriptional “on” state. P < 0.0001. Dotted lines represent quantiles; thick lines indicate medians. Statistical analysis was performed using an unpaired, two-tailed t-test in GraphPad Prism. (D) Schematic representation of the stem-loop feedback mechanism in NMD. (E) **E**xpected timing of transcriptional feedback. To estimate the expected timing, we evaluated the kinetics of mRNA from transcription to degradation. The transcription elongation rate ranges from 1.1 to 4.3 kb/min, with mRNA release typically completed within ~5 minutes for a ~4 kb transcript (29). Splicing can occur co-transcriptionally (30), the β-globin gene is relatively short, containing three exons and two introns. After transcription and maturation, mRNAs typically remain in the nucleus for ~5 minutes before export. Export is rapid, with nuclear pore complex (NPC) transit times of ~100–200 ms (31, 32). Once in the cytoplasm, mRNAs are generally targeted for translation within 2–5 minutes (33). Although the timing of degradation by NMD varies depending on mRNA context, for a PTC located at codon 39 of β-globin mRNA, recognition of the PTC can occur within approximately ~13 seconds (34–36). The entire process—from transcription to translation and subsequent mRNA decay—is expected to occur within ~10 minutes (26, 37)

Transcriptional activity was assessed by analyzing bursting dynamics, which reflect the timing and frequency of RNA polymerase II (Pol II) engagement at the transcription site (TS). We quantified both the frequency of TS bursting (Fig. 5A–B) and the duration of ON-states (Fig. 5A, C). While the burst frequency was comparable between the wild-type and PTC-containing alleles, indicating that transcription initiation occurs at similar rates, the ON-state durations were significantly prolonged in the PTC-containing allele. To interpret this phenomenon, we developed a minimal stochastic model that captures key features of the observed transcriptional dynamics (Fig. S9). The model simulates ON-OFF transitions in fluorescence intensity using a set of state-dependent stochastic differential equations. Crucially, we introduced a mechanism for self-regulation in which bursts are temporally correlated, resulting in progressively longer ON durations. This extension recapitulates the experimental time series observed for the PTC-containing allele and provides a conceptual basis for understanding the altered dynamics. These results suggest that the PTC mutation does not impact the frequency of Pol II recruitment but instead affects the kinetics of transcriptional progression. The increased ON-state duration may reflect delayed termination or Pol II release, potentially due to the retention of nascent RNA fragments targeted by NMD. Our findings support a model in which the presence of a PTC triggers a feedback mechanism that prolongs the transcriptional engagement of Pol II, potentially reinforcing gene regulation at the level of elongation or transcript clearance. Based on this, we propose a model of transcriptional feedback triggered by NMD (Fig. 5D). Our data showed that transcriptional enlargement became apparent approximately one hour after the transcription site, which was first detected using the PonA-inducible system. Based on previously reported mRNA processing kinetics, the entire process from transcription to NMD-mediated decay is expected to occur as early as 30 minutes after transcription initiation. This suggests that the feedback mechanism can begin within that time window (Fig. 5E).

We also observed that the transcriptional enlargement is transient, eventually diminishing over time (on average, within 1–2 hours, depending on the extent of the enlargement). This may be attributed to temporal stalling of RNA polymerase II (Pol II), which would lead to reduced release of mRNA transcripts. As a result, downstream processes such as export, translation, decay, and subsequent feedback would also be diminished. Once the feedback weakens, the release of nascent mRNA likely returns to its normal rate.

## Discussion

Growing evidence suggests a strong link between RNA turnover and transcription (1, 22–24). However, studying sequence-specific transcriptional feedback in NMD has been technically challenging due to the difficulty of linking nuclear with cytoplasmic events. In this study, we used simultaneous real-time imaging to detect transcriptional activities at both the wild-type and PTC-containing gene alleles, achieving high spatiotemporal resolution. Our findings provide robust evidence for rapid feedback from cytoplasmic decay to transcription in the nucleus. We also captured dynamic changes in the transcription site of the PTC-containing gene allele, driven by transcriptional stalling, which led to a stretched chromatin structure.

The phenomenon of transcriptional enlargement in NMD was first discovered using single-molecule fluorescence in situ hybridization (smFISH)(6) to address a paradox: despite the requirement for translation, the reduction of PTC-containing mRNA was detected in isolated nuclear fractions. While it was later confirmed that NMD occurs on the cytoplasmic side, immediately after mRNA export (25), this paradox remains unresolved. Our study suggests that transcriptional stalling at the site of the PTC-containing gene may explain the reduction of PTC-containing mRNA in nuclear fractions, as it is likely due to pre-mRNA stalling rather than mRNA decay in the nucleus.

One model proposed to explain transcriptional feedback in NMD involves the recognition of specific transcription sites by functional messenger ribonucleoprotein (mRNP) fragments that enter the nucleus. This possibility was previously explored by injecting in vitro synthesized uncapped RNA, but the results were inconclusive (8, 9). In our study, the use of a self-cleaving hammerhead ribozyme failed to trigger transcriptional enlargement, suggesting that RNA fragments alone are not sufficient to induce transcriptional enlargement. Additionally, the fact that nuclear import inhibition diminished the transcriptional enlargement of PTC-containing transcripts further supports the idea that a specific protein regulator may be involved in this mechanism.

Although RNA fragments generated by NMD are generally undetectable by biochemical methods such as northern blotting unless Xrn1 knockdown (19, 20), this suggests that most intermediate fragments are rapidly and efficiently degraded. However, a previous study using live-cell imaging to analyze the decay kinetics of 3′ fragment degradation by Xrn1 during NMD reported that Xrn1 occasionally dissociates from the RNA before completing degradation, implying that multiple rounds of Xrn1 engagement may be required for full transcript degradation (26). Such incomplete degradation could lead to the production of decay intermediate fragments, which may underlie a key mechanism by which these fragments regulate transcription. Notably, our results demonstrated that NMD-mediated transcriptional feedback is dependent on nuclear import of proteins, indicating the involvement of protein factors in the mechanism. One potential candidate is Xrn1 itself, which has been implicated in linking RNA decay to transcriptional regulation in previous studies (1, 2, 4, 5). Another is UPF1, which is known to associate with chromatin (10) and may play a role in this feedback loop. However, dissecting the specific contribution of these factors is challenging, as knockdown of Xrn1 or UPF1 not only disrupts the feedback mechanism but also impairs NMD activity itself. Therefore, further studies using refined experimental strategies will be required to identify the key protein factors that mediate NMD-coupled transcriptional feedback.

We propose that RNA molecules may have the potential to recognize specific genomic loci through RNA/DNA base pairing. One example of small RNA molecules regulating genomic DNA is the CRISPR-Cas system, which uses short (~20 base pairs) RNA molecules to identify and cleave specific genomic loci. Additionally, small double-stranded RNAs, such as those involved in RNA interference (RNAi) pathways (e.g., small interfering RNAs (siRNAs), PIWI-interacting RNA (piRNA)), are known to regulate chromatin modifications (27, 28), suggesting a common mechanism for small RNAs in chromatin regulation. Further studies are needed to elucidate the molecular mechanisms underlying these processes.

## Supplementary Material

Supplement 1 Materials and Methods
Supplementary Text
Figs. S1 to S9
References (1–6)
Movies S1 to S2

Supplement 2

Supplement 3

## Data Availability

The data and analysis code generated in this study are available from the corresponding authors upon reasonable request.
