# Supplementary material for: Real-time imaging of transcriptional feedback in nonsense-mediated mRNA decay
Source: bioRxiv. 2025 May 21:2025.05.20.655238. Preprint. [Version 1] doi: 10.1101/2025.05.20.655238 (PMC12139809; doi:10.1101/2025.05.20.655238)
Supplement: Supplement 1 — Materials and Methods Supplementary Text Figs. S1 to S9 References (1–6) Movies S1 to S2 [file media-1.pdf]

Supplementary Materials for

Real-time imaging of transcriptional feedback in nonsense-mediated mRNA decay

Hanae Sato[1,2*], Islam Md. Dobirul[1], and Tamoghna Das[1], Robert H. Singer[2]

Correspondence to: hanae-sato@staff.kanazawa-u.ac.jp

This PDF file includes:

Materials and Methods
Supplementary Text
Figs. S1 to S9
Captions for Movies S1 to S2

Other Supplementary Materials for this manuscript include the following:

Movies S1 to S2

Materials and Methods

Cell lines and tissue culture

The human U2OS FRT cell line was established in a previous study (*1*). U2OS FRT PonA cell line was generated by stable transfection with the pERV3 plasmid, which expresses the synthetic VP16-glucocorticoid/ecdysone receptor (VgEcR) and retinoid X receptor (RXR), both required for activation of transcription from the PonA promoter, as previously described (*2*). U2OS FRT PonA cell lines stably expressing the bi-directional PonA constructs pFRT-PonA-BI-Gl-WT-24xMS2-WT-18xPP7 (WW) and pFRT-PonA-BI-Gl-WT-24xMS2-PTC-18xPP7 (WP) were established in a previous study (2). In this study, additional stable cell lines were generated by co-transfection with pOG44 and selection with hygromycin. These include lines expressing pFRT-PonA-BI-Gl-WT-24xMS2-HHR-18xPP7 (WH), pFRT-PonA-BI-mu-WT-24xMS2-WT-18xPP7 (mu-WW), pFRT-PonA-BI-mu-WT-24xMS2-PTC-18xPP7 (mu-WP), pFRT-PonA-BI-Gl-WT-4xMS2exon-WT-5xPP7exon (WWex), and pFRT-PonA-BI-Gl-WT-4xMS2exon-PTC-5xPP7exon (WPex).

All cell lines were maintained at 37 °C with 5% CO_2 in Dulbecco's Modified Eagle Medium (DMEM) supplemented with 4.5 g/L glucose, 10% fetal bovine serum (FBS), and 1% penicillin-streptomycin.

Live cell imaging acquisition

For live-cell imaging, the culture medium was replaced with L-15 medium supplemented with 10% fetal bovine serum (FBS) and 1% penicillin-streptomycin prior to imaging. Wide-field fluorescence images were acquired using either an IX-81 inverted microscope (Olympus), equipped as previously described (3), or an ECLIPSE Ti2-E inverted microscope (Nikon) equipped with a four-laser unit (LUD-H4) and an ORCA-Fusion BT CMOS camera (Hamamatsu). The Olympus system was operated with MetaMorph software using a 60×/1.4 NA oil immersion objective, while the Nikon system was controlled by NIS-Elements software and used a CFI Apo TIRF 60XC Oil objective (MRD01691). During imaging, cells were maintained at 37 °C using a stage-top incubator (INUBH-ZILCS-F1, Tokai Hit, Japan).

Optical sectioning was performed using a 500 nm Z-step over a total depth of 5.0 μm. The exposure time was set to 50 milliseconds for each optical plane and channel. To inhibit translation, nonsense-mediated mRNA decay (NMD), or nuclear import of proteins, cells were treated with

cycloheximide (CHX, Tocris Bioscience, Cat# 0970) at 100 μg/ml, NMDI14 (Sigma-Aldrich, Cat# SML1538) at 40 μM, or importazole (Sigma-Aldrich, Cat# 401105) at 50 μM, according to the treatment timelines described in the corresponding figure legends.

UPF1 knockdown

UPF1 knockdown was performed using UPF1-targeting shRNA and control shRNA, as described previously(*3*). U2OS FRT PonA WW cells were transfected using a laboratory-prepared PEI-based transfection reagent (referred to as PEX-max). Cells were seeded at approximately 70–80% confluency in complete growth medium one day prior to transfection. For each well of a 6-well plate, plasmid DNA was diluted in 100 μL of Opti-MEM (Thermo Fisher Scientific), and 6 μL of PEX-max solution was added. The DNA and reagent mixture was incubated at room temperature for 15 minutes to allow complex formation, then added dropwise to the cells. Transfected cells were maintained at 37 °C with 5% CO_2 and harvested 48 hours post-transfection for analysis.

Real-time RT-qPCR

Total RNA was purified using either TRIzol™ Reagent (Thermo Fisher Scientific, Cat# 15596018) or TRI Reagent (Cosmo Bio, Cat# TR118), following the manufacturers' instructions. For reverse transcription PCR (RT-PCR), RNA was treated with RNase-free DNase I (1 U/μL; Thermo Fisher Scientific, Cat# EN0521) for 30 minutes at 37 °C. cDNA was synthesized from 0.2 μg of total RNA in a 25 μL reverse transcription reaction using SuperScript III Reverse Transcriptase (Invitrogen) with random hexamers, or using the GeneAce cDNA Synthesis Kit (Nippon Gene, Cat# 319-08881), according to the respective manufacturer's protocol.

Quantitative PCR (qPCR) was performed using either PowerUp SYBR Green Master Mix (Thermo Fisher Scientific, Cat# A25741) or PowerTrack™ SYBR Green Master Mix (Thermo Fisher Scientific, Cat# A46109). Relative gene expression levels were calculated using the ΔΔCt method, with beta-actin used as the housekeeping gene for normalization of UPF1 knockdown. All reactions were performed in technical triplicates, and results are presented as mean ± SEM. Primer sequences are provided in the supplementary table.

Data analysis

Time-lapse images of transcription were obtained by generating maximum intensity projections from the z-series. Images were acquired at 2-minute intervals, with total imaging duration varying by experiment as indicated in the corresponding figure legends. Transcription sites were detected and tracked using Trackmate (*4*). with the Simple LAP tracker. Fluorescence intensities of the transcription sites were measured and normalized to the diffusive EGFP or mScarlet signal in the nucleus. The normalized spot intensities at each time point were plotted and smoothed using R. Transcription burst peaks were identified using the *findpeaks* function in R.

The statistics of burst events, such as their duration, frequency, the intensity time-series was gathered after further analysis as follows. The signal (intensity time-series), sans the outliers, was smoothened first using a Savitzky-Golay filter. For both cases, with and without PTC, a polynomial of order 3 with a time-window around 40 frames produced reasonable smoothing. Defining an ON-state above a certain Intensity threshold, its duration and related statistics were computed. The bursts were identified as the local maxima of the original signals. The closely-spaced bursts (within three consecutive frames) are then replaced by their mean to reduce overestimation. The bursts so identified, the statistics of bursts separation, and burst frequency per hour are computed for all observed signals.

Simulation of self-regulated stochastic process

To model the observed transcriptional dynamics, we developed a simplified stochastic framework that captures the essential features of fluorescence intensity fluctuations seen in our experiments. In both wild-type (WT) and premature termination codon (PTC)-containing alleles, the fluorescence intensity exhibits stochastic transitions between an **OFF** state (characterized by a baseline intensity) and an **ON** state (characterized by elevated intensity). These ON and OFF states occur with randomly distributed durations. Notably, transcription from the PTC-containing allele displays significantly longer ON durations compared to WT.

Based on prior knowledge of transcriptional bursting, we describe the fluorescence intensity time series *I(t)* using the following stochastic differential equation:

$$dI(t) = \sigma_S dW_\tau^S + \alpha(t)\, I(t)dt \;-\; \lambda(t)\,[I(t) - I_{base}]\, dt.$$

Here, *I(t)* denote the observed fluorescence intensity. The first term represents a Wiener process $dW\tau^S$, where $S \in \{ON, OFF\}$ and σ_S denotes the noise amplitude associated with each state. The second term, active only during the ON state, represents an intensity growth process governed by

a time-dependent rate function α(t). The third term accounts for intensity decay toward a baseline value I_{base}, with a decay rate λ(t). During the OFF state, both α(t) and λ(t) are set to zero.

Although this stochastic model is a phenomenological approximation, it serves as a useful proxy for the underlying biochemical network, which is currently too complex to resolve using our experimental setup. However, our data suggest that this network may introduce correlations in ON-state duration, particularly evident in the transcriptional behavior of the PTC-containing allele.

To incorporate such correlations, we introduce a surrogate equation that governs the evolution of the ON-state duration τ_{ON}, conditioned on the change in fluorescence intensity:

$d\tau_{ON} = (\sigma_{\text{ON}} + \Delta\tau_{\text{ON}})\, dW_{\tau}^{ON}$ when $\Delta I > I_c$.

Here $\Delta I = I(t+1) - I(t)$, and I_c is a threshold value set arbitrarily. When $\Delta I > Ic$, the model introduces a small random increment $\Delta\tau_{\text{ON}}$, favoring prolonged ON durations in response to large intensity changes. This condition is based on the intuition that in a Wiener process, large changes between consecutive steps are rare and thus may encode weak correlations. Accordingly, $1/I_c$ can be interpreted as a proxy for the strength of this correlation.

This minimal stochastic description successfully recapitulates the key features of the transcriptional dynamics observed in our experimental data. Importantly, it provides a conceptual basis for understanding the mechanistic differences between WT and PTC-containing transcriptional activity.

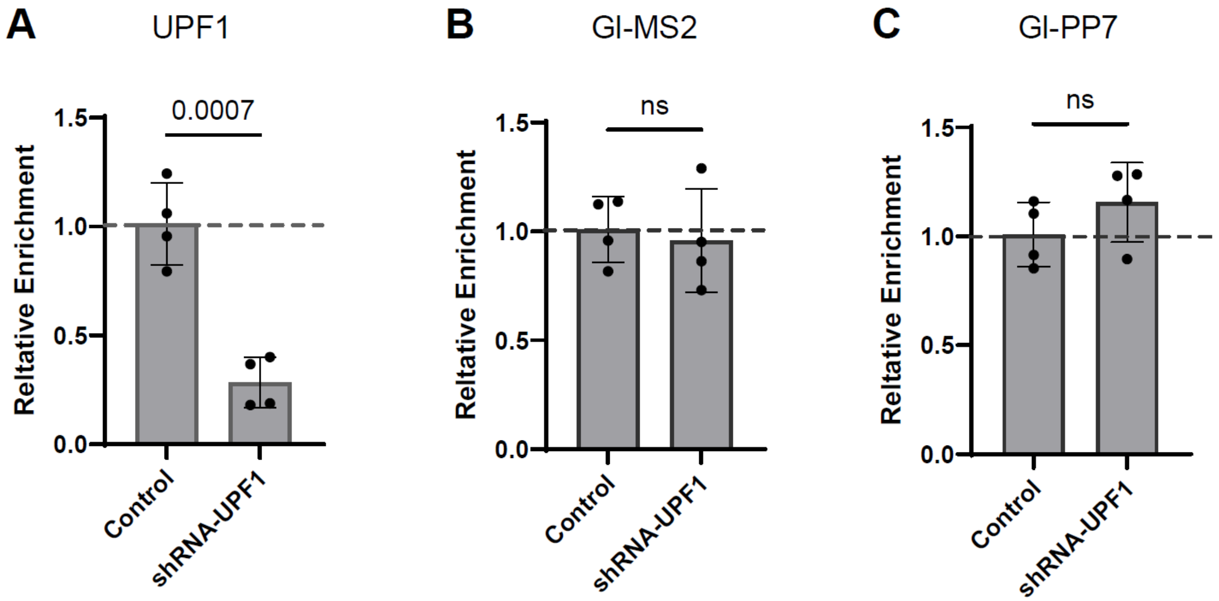

Fig. S1. Insertion of multiple stem loops into the 3'UTR does not induce NMD in the Gl reporter. (A) UPF1 knockdown using shRNA was confirmed by RT-qPCR. β-actin mRNA was used as a reference. P values were determined using two-tailed unpaired t-tests (P = 0.0007). (B) Quantitative detection of β-globin mRNA containing MS2 stem loops in WW-expressing cells was performed by RT-qPCR. (C) Quantitative detection of β-globin mRNA containing PP7 stem loops in WW-expressing cells was similarly performed. β-actin mRNA was used as a control. P values were determined using two-tailed unpaired t-tests (ns = not significant). Error bars represent standard deviation from three independent experiments. Statistical analysis was performed using GraphPad Prism software.

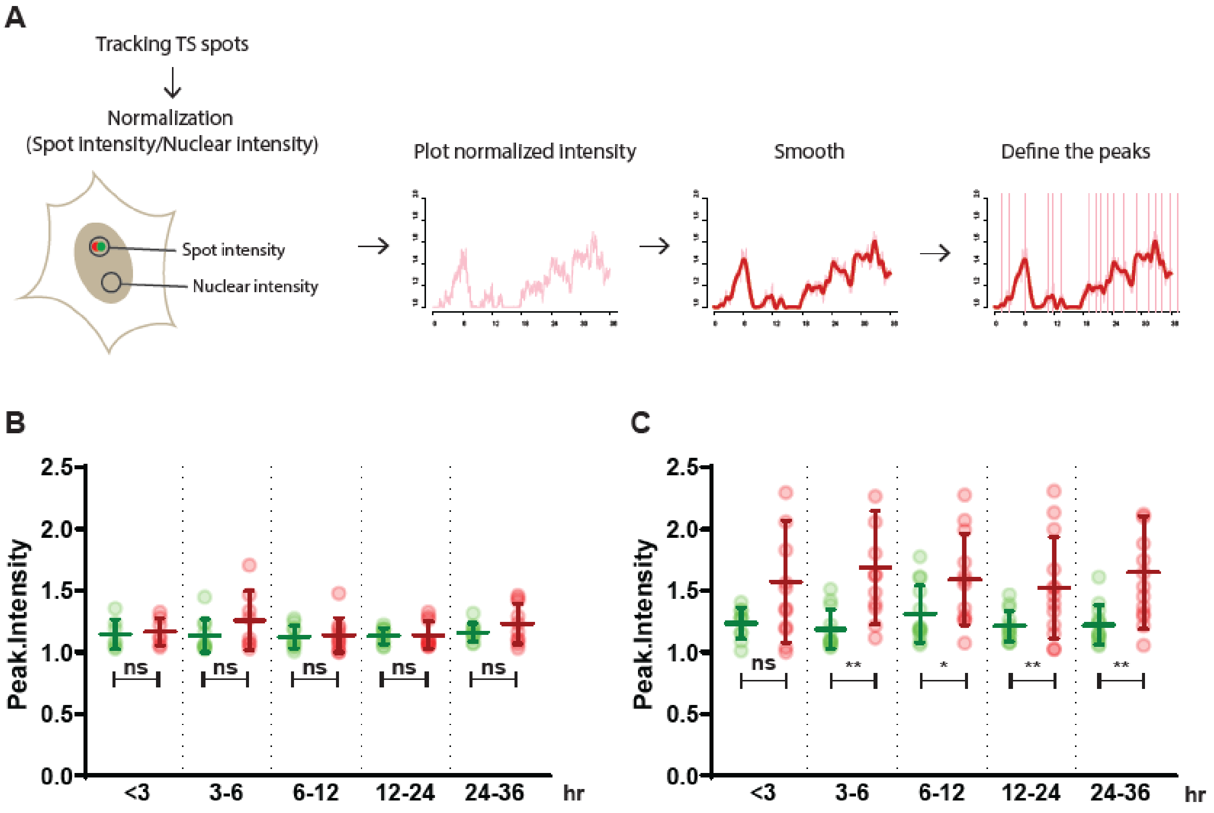

Fig. S2. Analytical scheme of peak detection and peak analysis of transcription sites. (A) Schematic of analytical scheme for transcriptional activity. Transcription sites bidirectionally expressing mRNA labeled with MS2 or PP7 system were imaged every 2-min for more than 36-hours in live cells and detected using TrackMate spot analysis software including the extension of TrackMate-extras for multi-channel tracking. Transcription sites labeled with green or red fluorescence proteins were tracked in the same ROI between each color except when transcription sites of each color expressing bi-directionally did not overlap. When one of the transcription sites expressing bidirectional was not detectable, a transcription site labeled with another color was used to define the ROI. When both transcription sites were temporally invisible, the action of "Close gaps by introducing new spots", which introduces the new spots based on the positions and size calculated using the linear interpolation from the track, was used. The mean intensity of identified spot at each time was normalized by the nuclear intensity and normalized intensities of transcription sites were plotted, smoothed using loess regression and smoothing in r. The peak (transcription burst) was defined using the findpeaks function in R (pracma: Practical Numerical Math Functions)(*5*). The code using this analysis is available upon request. Detection of

transcription sites expressing WW (B) or WP (C) construct for 36-hours. Single dots denote the mean of normalized intensity of transcription sites during indicated time duration from single cells. P values were determined using two-tailed unpaired t-tests (ns=not significant, **P<0.01, *P<0.05) from 10 (WW) or 13 (WP) cells. Error bars = Standard deviation in cell populations.

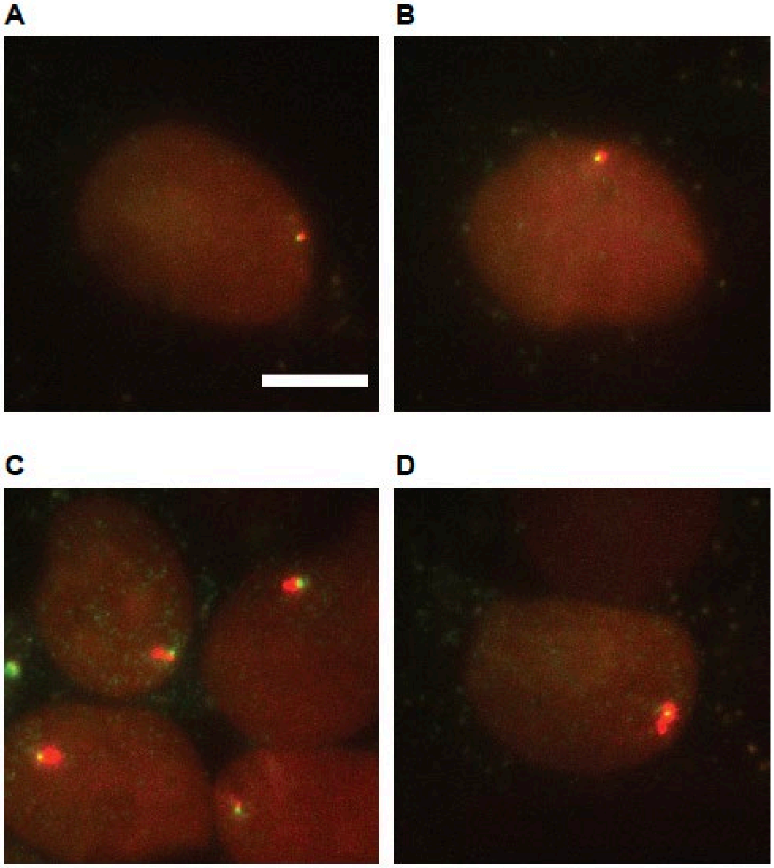

Fig. S3. Simultaneous detection of transcription sites at the Ig μ NMD reporter with and without PTC. The image of transcription site expressing wild-type Ig μ mini reporter mRNA from both directions (A) or, wild-type or PTC Ig μ mRNA from each direction of the promoter (B). Bar=10 μm.

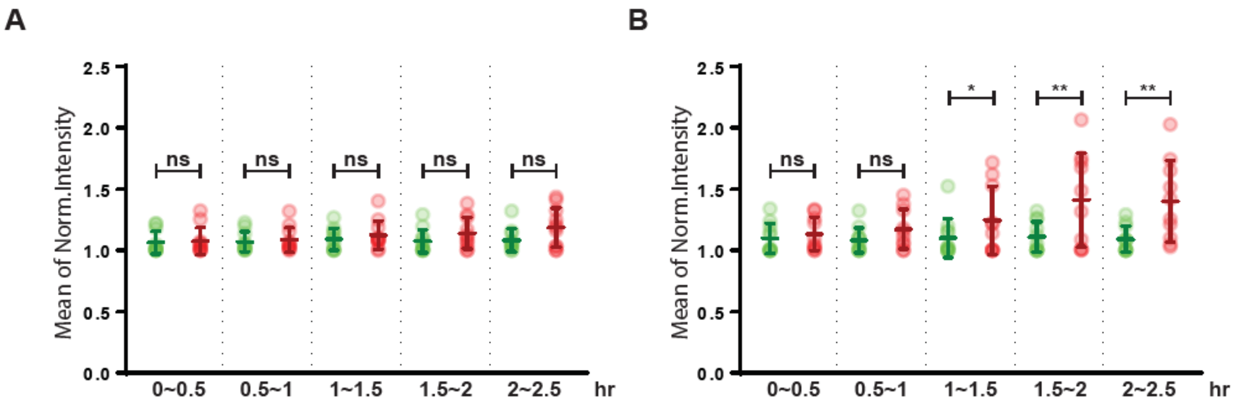

Fig. S4. Transcriptional enlargement begins about 1-hour after transcription was initiated. Detection of transcription sites expressing WW (A) or WP (B) construct. Single dots denote the mean of normalized intensity of transcription sites during indicated time duration from single cells. P values were determined using two-tailed unpaired t-tests (ns=not significant, **P<0.01, *P<0.05) from 11 (WW) or 10 (WP) cells. Error bars = Standard deviation in cell populations.

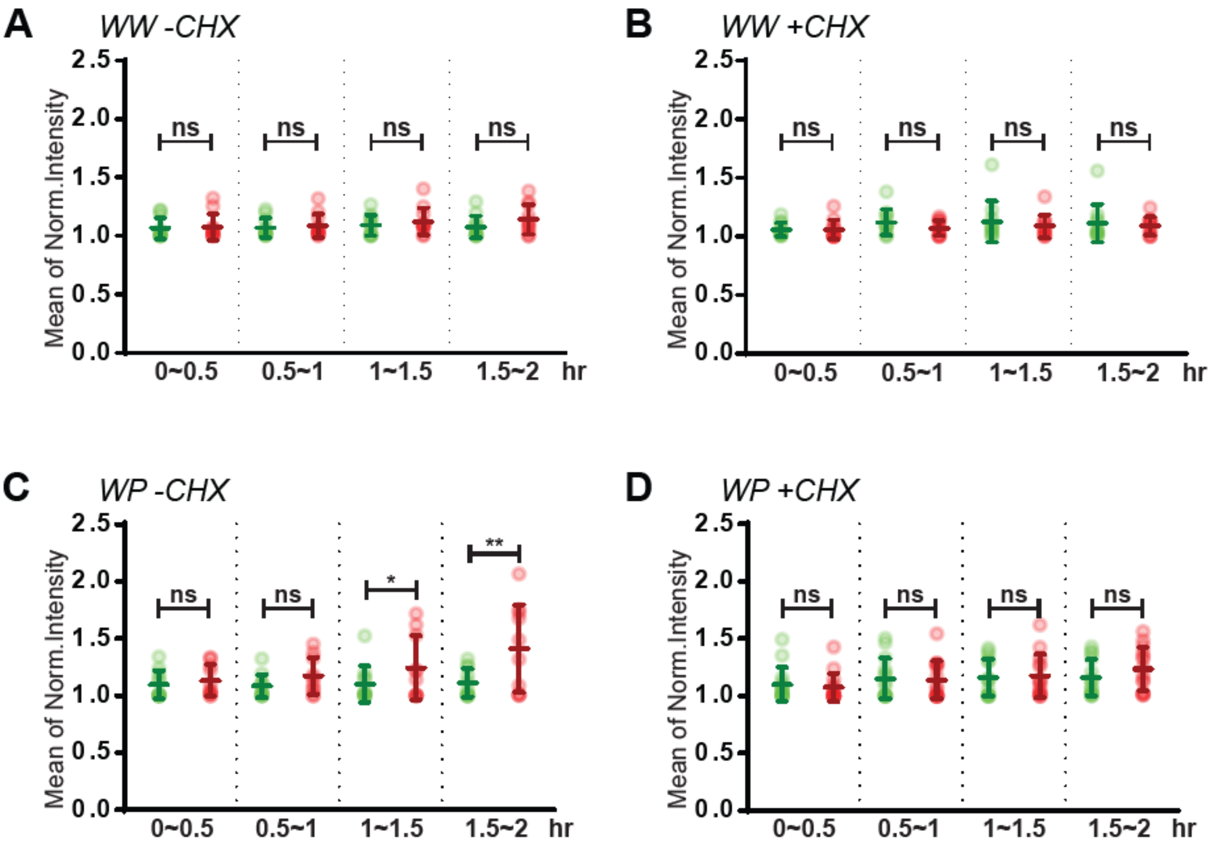

Fig. S5. Detection of transcription sites expressing the WW or WP construct with and without the translation inhibitor cycloheximide (CHX).

Detection of transcription sites expressing the WW construct without CHX (A) and with CHX (B), and the WP construct without CHX (C) and with CHX (D). Single dots represent the mean normalized intensity of transcription sites from individual cells (green: wild-type; red: PTC-containing β-globin transcription sites) during the indicated time intervals. P-values were determined using two-tailed unpaired t-tests (ns = not significant, $P < 0.05$, $P < 0.01$). Error bars indicate standard deviation across cell populations. N = 14.

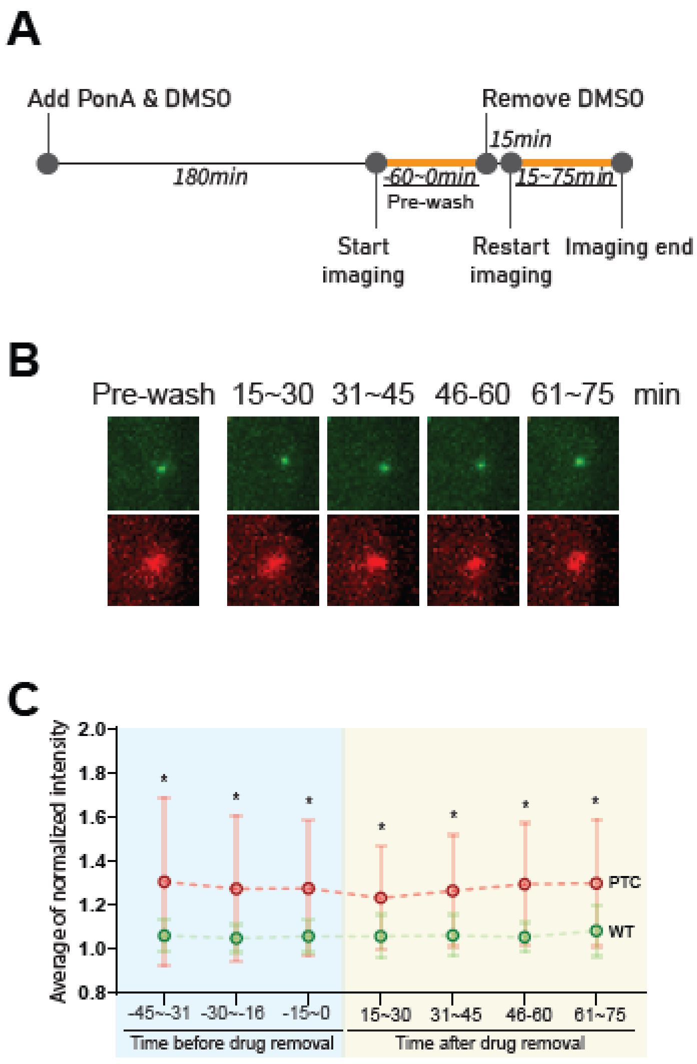

Fig. S6. Changing medium does not trigger PTC-specific transcriptional enlargement.

(A) The timeline of transcription induction by supplement of PonA with DMSO and following DMSO removal. Orange lines and underlined times indicate the time duration for real-time

imaging. (B) Images of transcription site before (Pre-wash) and after removal of DMSO (15-75 minutes). The image size of each transcription site shown here is 8 x 8 μm^2. (C) The detection of transcription activities expressing WP construct before (Pre-wash) and after removal of DMSO (10-90 min). The dots indicate the mean of averaged normalized intensity of transcription sites during indicated time duration in 13 cells. Error bars = Standard deviation in cell populations. The statistical analysis was performed by Graphpad prism software.

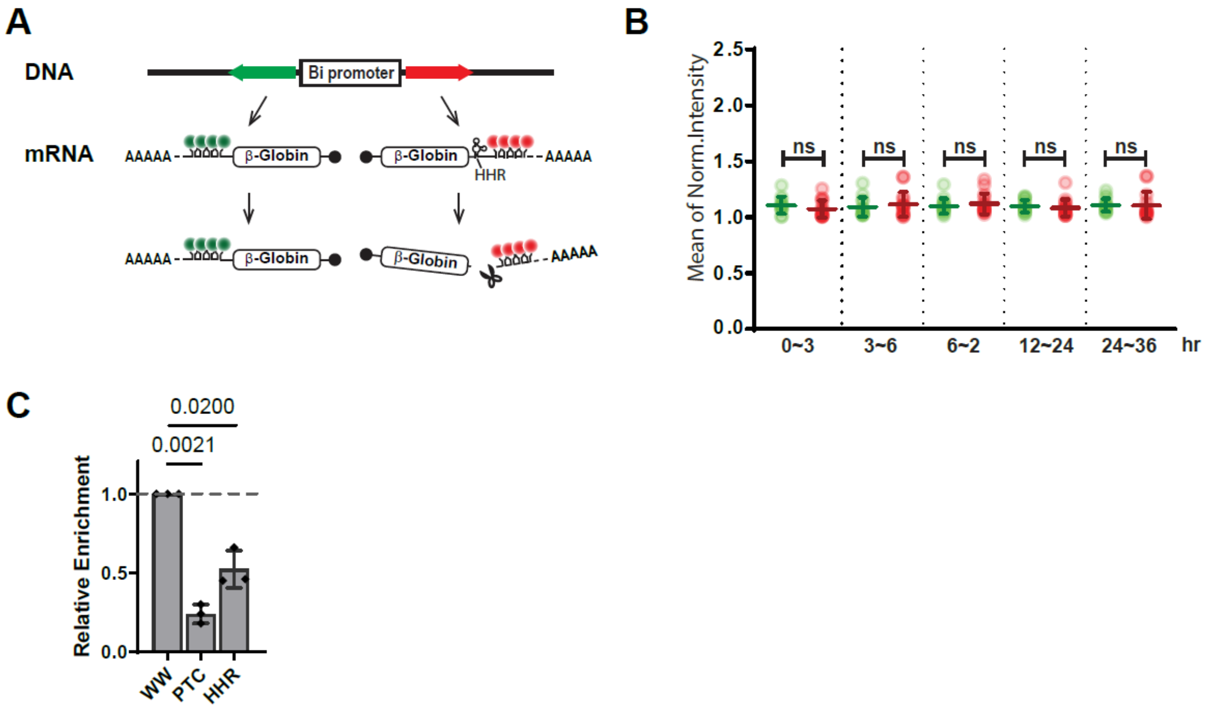

Fig. S7. mRNA self-cleavage with hammerhead ribozyme (HHR) does not trigger the transcriptional feedback. (A) Schematic of PonA inducible bi-directional promoter expressing wild-type β-globin genes with MS2 stemloops or with the self-cleaving motif of hammerhead ribozyme (HHR) between the β-globin ORF and PP7 stem loops. in the 3'UTR that labeled with stdMCP-stdGFP or stdPCP-stdmScarlet. (B) Simultaneous detection of transcription sites expressing wild-type with (Red) or without HHR (Green). Single dots denote the mean of normalized intensity of transcription sites in single cells during the indicated time duration. P values were determined using two-tailed unpaired t-tests (ns=not significant) from 15 cells. Error bars = Standard deviation in cell populations. (C) Quantitative detection of β globin mRNA with PP7 stem loops (Wild-type, PTC or HHR containing β globin expressing cells) were determined by RT-qPCR. MS2-tagged β globin mRNA (wild-type β globin mRNA in each cell) was used as a control. P values were determined using two-tailed unpaired t-tests (P=0.0021, P=0.02). Error bars = Standard deviation from three independent experiments. The statistical analysis was performed by Graphpad prism software.

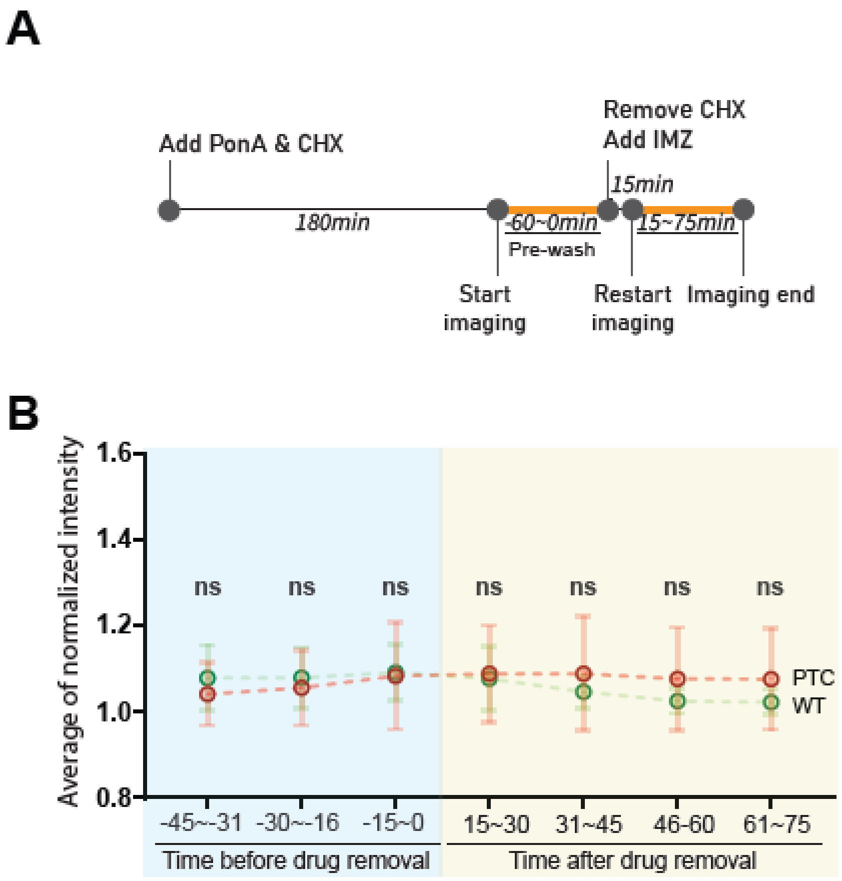

Fig. S8. Nuclear transport importin-β inhibitor, importazole inhibited PTC-specific transcription enlargement. (A) The timeline of transcription induction by supplement of PonA with translation inhibitor, CHX and following the removal of CHX and supplement of importin β inhibitor, 50 µM importazole (IMZ) (*6*). (B) The detection of transcription sites expressing WP construct before and after removal of CHX with the supplement of IMZ. Single dots denote the mean normalized intensity of wild-type (Green) or PTC-containing β-globin transcription sites (Red) during indicated time duration from 12 cells. Error bars = Standard deviation in cell populations. The statistical analysis was performed by Graphpad prism software.

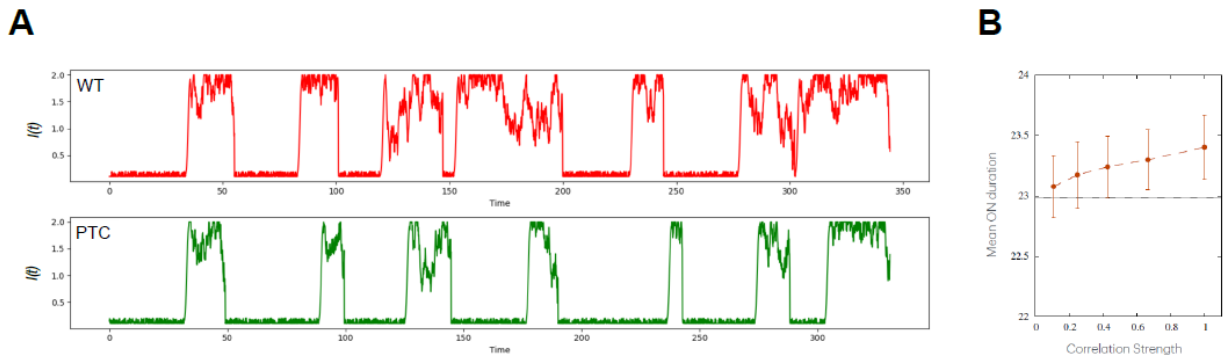

Fig. S9. Transcription as a self-regulated stochastic process: (A) Simulated time series of fluorescence intensity $\boldsymbol{I(t)}$ illustrating a single realization of the stochastic ON-OFF transcriptional process. The green trace represents the unregulated model, capturing the burst dynamics characteristic of the wild-type (WT) allele. The red trace demonstrates the effect of incorporating self-regulation via correlated burst durations, resulting in extended ON-state durations that resemble the transcriptional behavior observed for the PTC-containing allele. (B) The ON-state duration increases consistently with the strength of correlation, supporting the hypothesis that enhanced burst correlation underlies the prolonged transcriptional activity seen in the PTC context. This provides a conceptual explanation for the dynamic differences between WT and PTC-containing alleles, as discussed in the main text.

Supplementally table

Primer list for RT-qPCR

human_ACTB_F	TCCCTGGAGAAGAGCTACG
human_ACTB_R	GTAGTTTCGTGGATGCCACA
human_UPF1_F	AGATCACGGCACAGCAGAT
human_UPF1_R	TGGCAGAAGGGTTTTCCTT
GI3'_F	AGAAGGTGGTGGCTGGAG
MS2v5_R	CCGTTTGTAGGTTACCGG
PP7ORFv5_R	AAGAAGTGGATCCCATACC

Movie S1. (separate file)

Live imaging of the U2OS cell expressing WW. The transcriptions of reporter genes were induced using Ponasterone A. The wild-type β-globin transcripts expressing from either direction of promoter contains MS2 or PP7 sequences in the 3'UTR that was labeled with stdMCP-stdGFP or stdPCP-stdmScarlet as described in Figure 1. Each transcription site was imaged every 2 min.

Movie S2. (separate file)

Live imaging of the U2OS cell expressing WP. The transcriptions of reporter genes were induced using Ponasterone A. The wild-type or PTC-containing β-globin transcripts expressing from either direction of promoter contain MS2 or PP7 sequences in the 3'UTR that was labeled with stdMCP-stdGFP or stdPCP-stdmScarlet as described in Figure 1. Each transcription site was imaged every 2 min.

1. A. Senecal, B. Munsky, F. Proux, N. Ly, F. E. Braye, C. Zimmer, F. Mueller, X. Darzacq, Transcription factors modulate c-Fos transcriptional bursts. *Cell Rep* **8**, 75–83 (2014).

2. H. Sato, R. H. Singer, Cellular variability of nonsense-mediated mRNA decay. *Nat Commun* **12**, 7203 (2021).

3. W. Li, A. Maekiniemi, H. Sato, C. Osman, R. H. Singer, An improved imaging system that corrects MS2-induced RNA destabilization. *Nat Methods* **19**, 1558–1562 (2022).

4. J. Y. Tinevez, N. Perry, J. Schindelin, G. M. Hoopes, G. D. Reynolds, E. Laplantine, S. Y. Bednarek, S. L. Shorte, K. W. Eliceiri, TrackMate: An open and extensible platform for single-particle tracking. *Methods* **115**, 80–90 (2017).

5. H. W. Borchers, pracma: Practical Numerical Math Functions, (2011); https://doi.org/10.32614/CRAN.package.pracma.

6. J. F. Soderholm, S. L. Bird, P. Kalab, Y. Sampathkumar, K. Hasegawa, M. Uehara-Bingen, K. Weis, R. Heald, Importazole, a small molecule inhibitor of the transport receptor importin-beta. *ACS Chem Biol* **6**, 700–8 (2011).
